# VKNG-1 Antagonizes ABCG2-Mediated Multidrug Resistance via p-AKT and Bcl-2 Pathway in Colon Cancer: In Vitro and In Vivo Study

**DOI:** 10.3390/cancers13184675

**Published:** 2021-09-17

**Authors:** Silpa Narayanan, Ying-Fang Fan, Nehaben A. Gujarati, Qiu-Xu Teng, Jing-Quan Wang, Chao-Yun Cai, Yuqi Yang, Anirudh J. Chintalapati, Yixiong Lei, Vijaya L. Korlipara, Zhe-Sheng Chen

**Affiliations:** 1 Department of Pharmaceutical Sciences, College of Pharmacy and Health Sciences, St. John’s University, Queens, NY 11439, USA; silpa.narayanan16@my.stjohns.edu (S.N.); fanyf068700@sina.com (Y.-F.F.); nehagujarati@gmail.com (N.A.G.); qiuxu.teng15@my.stjohns.edu (Q.-X.T.); jingquan.wang16@my.stjohns.edu (J.-Q.W.); chaoyun.cai16@my.stjohns.edu (C.-Y.C.); yuqi.yang17@my.stjohns.edu (Y.Y.); anirudh.chintalapati16@my.stjohns.edu (A.J.C.); 2 Department of Hepatobiliary Surgery, Zhu Jiang Hospital of Southern Medical University, Guangzhou 510282, China; 3 School of Public Health, Guangzhou Medical University, Guangzhou 511436, China; gz-leizeng@163.com

**Keywords:** ATP binding cassette (ABC) transporter, MDR, reversal, tumor xenograft model, VKNG-1

## Abstract

**Simple Summary:**

Multidrug resistance or chemoresistance is a phenomenon where cells exhibit resistance to drugs that are pharmacologically and structurally distinct. ABCG2, a member of ABC transporter superfamily has been widely reported to be a principal cause of MDR in various cancers via pumping out various antineoplastic drugs. VKNG-1, a phenyltetrazole analogue selectively inhibits the ABCG2 transporter and reverses resistance to standard anticancer drugs both in vitro and in vivo in ABCG2-overexpressing colon cancers. This study presents the importance of VKNG-1 as a modulator of the ABCG2 transporter. In cancer patients, a combination of VKNG-1 and ABCG2 substrate drugs could be a beneficial treatment option for cells with high ABCG2 expression.

**Abstract:**

The emergence of multidrug resistance (MDR) to chemotherapeutic drugs is a major problem in the therapy of cancer. Knowledge of the mechanisms of drug resistance in cancer is necessary for developing efficacious therapies. ATP-binding cassette (ABC) transporters are transmembrane proteins that efflux chemotherapeutic drugs from cancer cells, thereby producing MDR. Our research efforts have led to the discovery of VKNG-1, a compound that selectively inhibits the ABCG2 transporter and reverses resistanctabe to standard anticancer drugs both in vitro and in vivo. VKNG-1, at 6 µM, selectively inhibited ABCG2 transporter and sensitized ABCG2-overexpressing drug-resistant cancer cells to the ABCG2 substrate anticancer drugs mitoxantrone, SN-38, and doxorubicin in ABCG2-overexpressing colon cancers. VKNG- 1 reverses ABCG2-mediated MDR by blocking ABCG2 efflux activity and downregulating ABCG2 expression at the mRNA and protein levels. Moreover, VKNG-1 inhibits the level of phosphorylated protein kinase B (PKB/p-AKT), and B-cell lymphoma-2 (Bcl-2) protein which may overcome resistance to anticancer drugs. However, the in vitro translocation of ABCG2 protein did not occur in the presence of 6 µM of VKNG-1. In addition, VKNG-1 enhanced the anticancer efficacy of irinotecan in ABCG2- overexpressing mouse tumor xenografts. Overall, our results suggest that VKNG-1 may, in combination with certain anticancer drugs, represent a treatment to overcome ABCG2-mediated MDR colon cancers.

## 1. Introduction

Cancer is the second main cause of death worldwide [1,2] and according to the World Health Organization, 1,762,450 new cases and around 606,880 cancer deaths have been disclosed in the United States [3,4]. Drug resistance in cancer cells can attenuate or abrogate the efficacy of anticancer drug treatment, thereby contributing to most cancer-related deaths [5,6,7,8,9,10,11,12]. Colorectal cancer (CRC) has been identified as a common form of cancer [13] and although the FOLFIRI regimen (folinic acid, 5-fluorouracil, and irinotecan) increases the survival rate of metastatic CRC patients, only approximately 50% of patients show an objective response because of resistance to the treatment [14]. One of the leading causes of drug resistance is the overexpression of ABCG2 transporter in metastatic CRC, and drugs such as SN-38 and its prodrug, irinotecan, are known substrates for the ABCG2 transporter [15]. The emergence of resistance of cancer cells to different chemotherapeutic drugs is defined as multidrug resistance (MDR) [16]. One of the reasons behind MDR is the high expression of ATP-binding cassette (ABC) transporters, which attenuate the effect of antineoplastic agents by actively extruding them from cancer cells [12,17]. These efflux pumps utilize ATP to alter the conformation of the ABC transporters to efflux endogenous and xenobiotic compounds from various cancer cells and are present in mammals and other microorganisms [18]. One of them, the ABCG2 transporter, which is 72 kDa and is classified as a half transporter and becomes active when it undergoes dimerization [19]. The ABCG2 transporter is present in the brain, gastrointestinal tract, testis, human placenta, and in the cancer cell line resistant to mitoxantrone, S1-M1-80 (leading to the term MXR) [20]. ABCG2 has been widely reported to be a principal cause of MDR in various cancers via pumping out various antineoplastic drugs such as tyrosine kinase inhibitors (TKIs), topoisomerase inhibitors, anthracyclines etc. [21]. Several studies have established that cancer stem-like cell (CSC) population in cancer expressing ABCG2 limits the efficacy of chemotherapy and is responsible for the reemergence of tumors during the period of relapse in various cancer patients. ABCG2 transporter is a target for possible clinical intervention as it expresses in normal tissues and its role in causing cancer drug resistance. In cancer therapeutics, a strategy of ABCG2 inhibition could be employed to increase bioavailability of chemotherapy agents and overcome cancer cell resistance [22].

The majority of MDR modulators are inhibitors of ABC transporters, which are administered prior to or simultaneously with the anticancer drug [23], can reverse drug resistance to certain anticancer drugs by binding to these transporters [24]. Recently, we reported the synthesis of a novel compound, VKNG-1 (N-(2-(2-(3-methoxyphenyl)-2H-tetrazol-5-yl) phenyl)-4-aminobenzamide) which is a phenyltetrazole analogue that contains amide linker [25]. The synthesis of the compound is already published in a previous study (VKNG-1 is named as compound 27) [25] and this compound is more potent than VKNG-2, a benzamide derivative and has a different mechanism for reversal of ABCG-2 mediated chemoresistance [26]. In our study, we chose to test whether VKNG-1 could act as a chemosensitizing agent to the ABCG2-overexpressing MDR phenotype of cancer cells and explore the mechanism of ABCG2-mediated chemoresistance.

Our results indicate that VKNG-1 reverses ABCG2-mediated MDR and an ideal candidate for combination therapy with conventional chemotherapy targeting ABCG2-mediated MDR. The phenyltetrazole ring and the amide at the R2 position in VKNG-1 are important for binding to ABCG2 and the -OCH3 group at the R1 position is involved in the inhibition of the ABCG2 transporter [27]. Therefore, in this manuscript, experiments are conducted to determine the reversal mechanism of VKNG-1 in anticancer drug resistance in vitro and in a tumor xenograft model in drug-resistant ABCG2-overexpressing colon cancers.

## 2. Results

### 2.1. VKNG-1 Increases the Sensitivity of ABCG2-Ooverexpressing Drug-Resistant Cells to Anticancer Drugs

**Figure 1:**
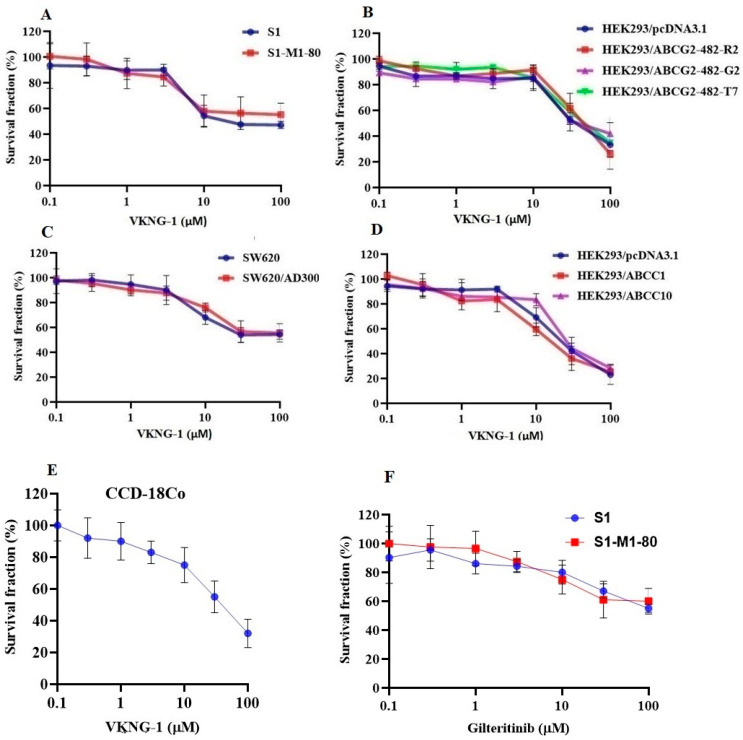
Cytotoxicity of VKNG-1 and gilteritinib on CCD-18Co, parental and drug-resistant cell lines. The S1 (blue) and S1-M1-80 (red) (**A**), HEK293/pcDNA3.1 (blue), HEK293/ABCG2-482-R2 (red), HEK293/ABCG2-482-G2 (violet) and HEK293/ABCG2-482-T7 (green) cell lines (**B**), SW620 (blue) and SW620/AD300 (red) cell lines (**C**) and HEK293/pcDNA3.1 (blue), HEK293/ABCC1 (red) and HEK293/ABCC10 (violet) cell lines (**D**) and CCD-18Co cell line (**E**) were treated with different concentrations of VKNG-1 (µM) for 72 h and S1 (blue) and S1-M1-80 (red) cells were incubated with gilteritinib (0-100 µM) for 72 h (**F**) and cell survival fraction (%) was measured. All data are shown as mean ± SD and represents three independent experiments.

The MTT assay was performed to determine the safe concentration (concentration at which 85% of cells survive) of VKNG-1 that could be used for the reversal experiments. From the cytotoxicity assay, 1 and 6 µM of VKNG-1 in both S1, S1-M1-80 (Figure 1A) and ABCG2-overexpressing transfected cells (Figure 1B) and 1 and 5 µM of VKNG-1 in SW620, SW620/AD300 (Figure 1C), HEK293/pcDNA3.1, HEK293/ABCC1 and HEK293/ABCC10 cells (Figure 1D) and 3 µM of gilteritinib were chosen for the reversal experiments (Figure 1F). VKNG-1, at 6 µM, like FTC, remarkably sensitized the mitoxantrone resistant S1-M1-80 cells (Table 1) and the transfected ABCG2 cell lines, HEK293/R482, HEK293/R482G and HEK293/R482T to the anticancer drugs mitoxantrone, SN-38 (active metabolic product of irinotecan) and doxorubicin (Table 2). The drug selected cell lines have characteristics like those in vivo but multiple factors that can cause MDR other than overexpression of the ABCG2 transporter [28] and the role of ABCG2-mediated resistance can be confirmed using transfected cell lines, although these cells are non-cancerous. Compared to the S1 cancer cell line, the resistant-fold values of the drug-resistant cancer cells to the ABCG2 specific substrate antineoplastic drugs mitoxantrone, SN-38 and doxorubicin, were significantly reduced by 1.6, 2.2 and 0.5-fold, respectively (Table 1). In addition, VKNG-1, at 6 µM, completely reversed the resistance to mitoxantrone, SN-38 and doxorubicin in the ABCG2 transfected cell lines (Table 2). The reversal efficacy of VKNG-1 for the ABCG2 transporter was similar to a known ABCG2 inhibitor (FTC) [29] at 6 µM. Moreover, VKNG-1 could not remarkably sensitize the parental cells to ABCG2 substrate anticancer drugs, and it had no significant effect on the efficacy of cisplatin (Table 1 and Table 2), an anticancer non-substrate drug for the ABCG2 transporter [30]. PI3K/AKT signaling leads to reduced apoptosis, stimulates cell growth and increases proliferation. In most tumors, PI3K/AKT hyper-activation are observed at a greater rate. In colorectal cancer, the most observed pathway changes are IGF2 overexpression and KRAS mutation which are located upstream in the PI3K/AKT pathway and activates them. Some drugs can specifically modulate the PI3K/AKT network, but other alternative pathways are of utmost importance and can modulate chemoresistance to drugs such as cisplatin that explains the treatment of drug-resistant cells with VKNG-1 do not increase the sensitivity to cisplatin even though VKNG-1 significantly decreased p-AKT activation [31].

Our results show that VKNG-1 can reverse MDR in both wild-type and mutant ABCG2-overexpressing cell lines. For SW620 (parental), SW620/AD300 (resistant) and HEK293/pcDNA3.1 (parental), and resistant cells, HEK293/ABCC1 and HEK293/ABCC10, 1 and 5 µM of the test compound was used. However, VKNG-1 did not significantly reverse MDR in SW620/AD300, that overexpress ABCB1 (Table 3), HEK293/ABCC1 (Table 4) and HEK293/ABCC10-overexpressing cell lines (Table 5) compared to the positive controls, verapamil, MK-571 and cepharanthine respectively. The rationale of testing the reversal effect of VKNG-1 on these cell lines is because the ABCC subfamily (includes ABCC1 and ABCC10 transporters), commonly called the multidrug resistance protein (MRP) family, has been shown to be associated with MDR in various cancers, including lung cancer (both small and non-small cell lung cancers), bladder cancer and breast cancer [32]. To find out the toxicity profile of VKNG-1 at higher doses, a cytotoxicity assay was carried out in human fibroblast cell line, CCD-18Co cell line (Figure 1E) and it is non-toxic even at high doses.

### 2.2. VKNG-1 Significantly Enhances the Accumulation and Diminishes the Efflux of (^3^H)-Mitoxantrone in Cells Overexpressing the ABCG2 Transporter

**Figure 2:**
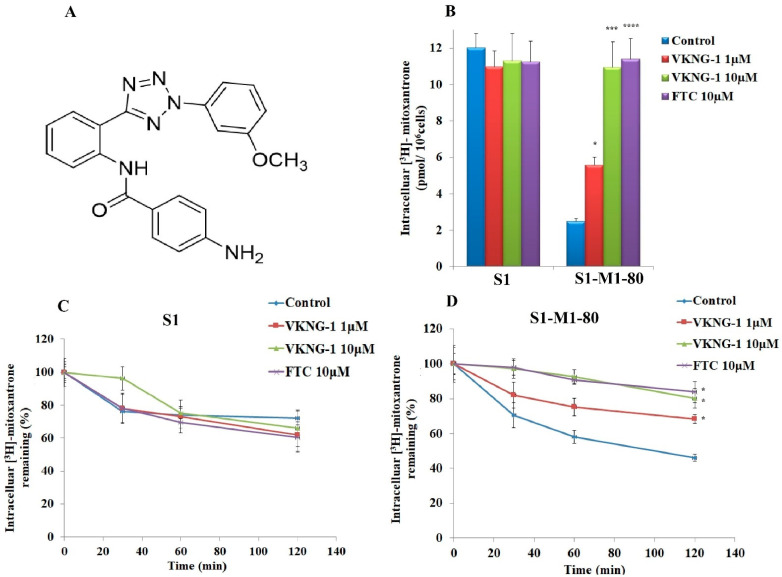
Chemical structure of VKNG-1 (**A**). The outcome of VKNG-1 activity on the cellular accumulation of (^3^H)-mitoxantrone in S1 and S1-M1-80 cells (**B**). Data of VKNG-1 on the efflux of the substrates of ABCG2. A graph was plotted with time course (0, 30, 60, 120 min) on the *X*-axis versus the percentage of intracellular (^3^H)-mitoxantrone remaining (%) on the *Y*-axis and showed the effect of VKNG-1 in S1 (**C**), S1-M1-80 (**D**) cells. Columns depict triplicate determinations; the error bars represent the SD. FTC at a concentration of 10 μM is used as an ABCG2 positive control inhibitor. * *p* < 0.05 and *** *p* < 0.001 compared to the control group. If *p*-value < 0.05, it is statistically significant and **** *p* < 0.001 is considered to be statistically highly significant.

The structure of VKNG-1 (N-(2-(2-(3-methoxyphenyl)-2H-tetrazol-5-yl) phenyl)-4-aminobenzamide) is shown in Figure 2A. To study the reversal mechanisms of VKNG-1, drug accumulation and efflux assays were carried out using the radioactive (^3^H)-mitoxantrone in the sensitive, S1 and S1-M1-80 (drug-resistant) cells. The intracellular level of mitoxantrone was low in drug-resistant cells when they were cultured with mitoxantrone alone; however, VKNG-1, at 10 µM increased the accumulation of (^3^H)-mitoxantrone in the S1-M1-80 cells significantly. In contrast, VKNG-1 did not significantly change the accumulation of (^3^H)-mitoxantrone in the parental cells (Figure 2B). These data show that VKNG-1 can rise (^3^H)-mitoxantrone accumulation in ABCG2-overexpressing cells.

A time-course experiment was performed to assess the efflux function of the ABCG2 transporter by estimating the level of (^3^H)-mitoxantrone remaining in the cells either in the presence or absence of VKNG-1 or FTC, an inhibitor of the ABCG2 transporter to further characterize the VKNG-1-induced rise in the intracellular level of (^3^H)-mitoxantrone in MDR cells. In the S1-M1-80 cells which are incubated with vehicle, the remaining intracellular (^3^H)-mitoxantrone levels after 30, 60 and 120 min of incubation, were 70%, 58% and 46%, respectively However, the treatment of the S1-M1-80 cancer cells with 10 µM of VKNG-1 notably increased the intracellular accumulation of (^3^H) mitoxantrone (Figure 2B). In contrast, VKNG-1, compared to the vehicle, did not notably modify the efflux of (^3^H)-mitoxantrone from the parental cancer cells, which lack the ABCG2 transporter overexpression (Figure 2C). Furthermore, like VKNG-1, the incubation of drug-resistant cancer cells with 10 μM of FTC, significantly decreased (^3^H)-mitoxantrone efflux in a time-dependent manner (Figure 2D).

Overall, our results indicated that VKNG-1 significantly enhanced the intracellular accumulation of (^3^H)-mitoxantrone by blocking the ABCG2 transporter’s efflux activity.

### 2.3. Effect of VKNG-1 and Gilteritinib on the Expression of ABCG2

**Figure 3:**
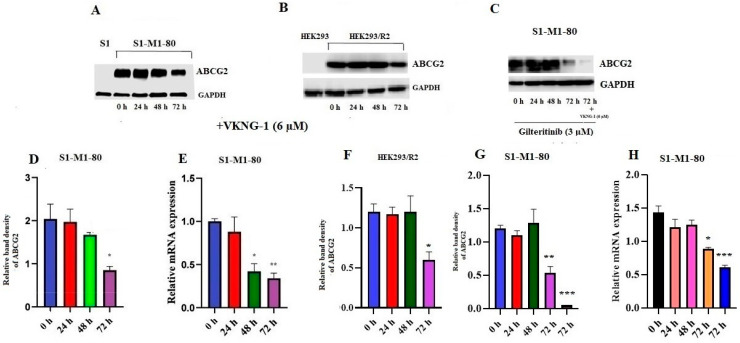
Effect of VKNG-1 and gilteritinib on the expression of ABCG2: The relative intensity of ABCG2 in S1-M1-80 cells (**A**), HEK293/R2 cells (**B**) was tested after the cells were incubated with 6 μM of VKNG-1 for 24, 48 and 72 h and in S1-M1-80 cells (**C**) after they were incubated with 3 μM of gilteritinib for 24, 48 and 72 h and with a combination of 6 μM of VKNG-1. Relative quantification of the effect of VKNG-1 on ABCG2 in S1-M1-80 cells (**D**) HEK293/R2 (**F**) and the effect of combination of VKNG-1 and gilteritinib on the expression of ABCG2 in S1-M1-80 cells (**G**). The expression level of the target protein (ABCG2) was normalized to GAPDH. Equal amounts of total cell lysates were employed for each sample and Western blot analysis was conducted. The effect of VKNG-1 (**E**) and the combination of VKNG-1 and gilteritinib (**H**) on the expression of ABCG2 in S1-M1-80 cells at mRNA level, normalized to the house keeping gene, 18S. * *p* < 0.05, ** *p* ≤ 0.01 and *** *p* ≤ 0.001 compared to the control group.

It is established that the reversal of MDR by a compound could result from the blockade of the efflux activity and/or by the downregulation of the ABCG2 transporter [33]. Therefore, we also conducted experiments to determine the expression of the ABCG2 transporter protein using Western blotting. The incubation of drug selective S1-M1-80 cancer cells (Figure 3A,D) and ABCG2 transfected HEK293/R2 cells (Figure 3B,F) with 6 µM of VKNG-1 for 72 h remarkably decreased the ABCG2 protein expression compared to cells incubated with vehicle.

To find out the association between p-AKT and ABCG2 down-regulation by VKNG-1 on S1-M1-80 cells, we evaluated the effect of VKNG-1 on ABCG2 protein and mRNA expression in presence of a p- AKT inhibitor, gilteritinib. The incubation of S1-M1-80 cancer cells with gilteritinib at a concentration of 3 µM (from the cytotoxicity data) for 24, 48 and 72 h and a combination of 3 µM of gilteritinib and 6 µM of VKNG-1 for 72 h remarkably decreased the ABCG2 expression at transcription and translational levels compared to cells incubated with vehicle (Figure 3C,G,H).

Furthermore, RT-PCR (quantitative real-time PCR) experiments demonstrated that the treatment of S1-M1-80 cells with 6 µM of VKNG-1 for 72 h remarkably decreased ABCG2 mRNA expression (Figure 3E).

These results showed that the downregulation of ABCG2 by VKNG-1 occurs at the pre-transcriptional level. Hence proved that the reversal effect of ABCG2-mediated MDR by VKNG-1 is mainly by 1) inhibition of the efflux activity of the ABCG2 transporter 2) downregulation of the ABCG2 mRNA and protein. Further experiments must be conducted to delineate how VKNG-1 downregulates mRNA and protein expression.

### 2.4. Effect of VKNG-1 on the Membrane Localization of ABCG2 Transporter

**Figure 4:**
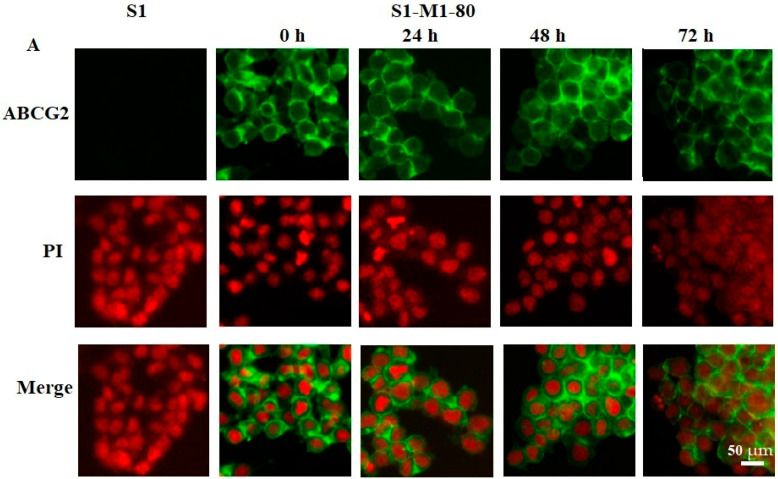
The effect of VKNG-1 on the expression and localization of ABCG2. S1 and S1-M1-80 cells were incubated with 6 µM of VKNG-1 for 24, 48, 72 h, respectively. The green color represents the ABCG2 transporter, and the red color represents the nucleus. The scale bar is 50 µm.

Altering the subcellular localization (i.e., the ABCG2 protein is not localized in the cytoplasmic cell membrane) of the ABCG2 transporter by VKNG-1 could also be involved in its reversal efficacy. Therefore, we conducted an immunofluorescence experiment to find out if VKNG-1 altered the localization of the ABCG2 protein from the cell surface to the cytosol. Our results indicated that incubating S1-M1-80 cancer cells with 6 µM of VKNG-1 for a time point of 72 h did not induce the translocation of the ABCG2 transporter from the cell membrane to the cytosol (Figure 4).

### 2.5. Docking Analysis of VKNG-1 and the Human ABCG2 Model

**Figure 5:**
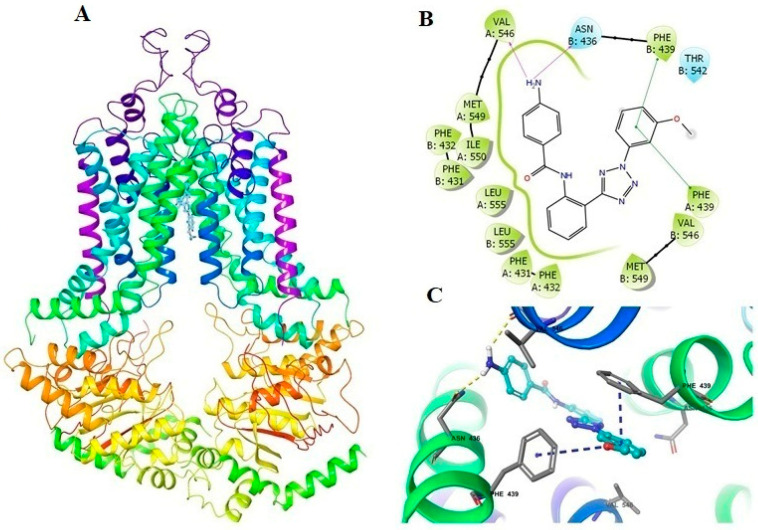
The molecular modeling of VKNG-1 and human ABCG2. The binding site of VKNG-1 within human ABCG2 (**A** and **B**). The two-dimensional diagram of VKNG-1 and human ABCG2 was shown. The residues within 3 Å are represented as colored bubbles: cyan—polar, green—hydrophobic. The purple arrows show hydrogen bonds and green short lines indicate π-π interactions (**C**). Docked position of VKNG-1 within the binding site of ABCG2. VKNG-1 is shown as ball and stick mode with the atoms colored: carbon—cyan, nitrogen—blue, oxygen—red, hydrogen—white. Important residues are in the form of sticks with grey color. Hydrogen bonds and π-π stacking interactions are indicated with yellow and cyan dotted short line, respectively.

The best-scored positions of the VKNG-1 for the human ABCG2 protein is displayed in Figure 5A, with a binding score of −12,929 kcal/mol. The binding of VKNG-1 to the ABCG2 protein involved hydrogen bonds and π-π interactions. The methoxyphenyl ring of VKNG-1 had π-π interactions with Phe439 in the A and B chains. The amino group of VKNG-1 formed two hydrogen bonds with Asn436 and Val546. VKNG-1 was bound in the hydrophobic pocket formed by Phe431, Phe432, Val546, Met549, Ile550 and Leu555 in the drug-binding pocket of ABCG2 (Figure 5B,C).

### 2.6. Effect of VKNG-1 on p-AKT and B-cell lymphoma-2 (Bcl-2) Expression

**Figure 6:**
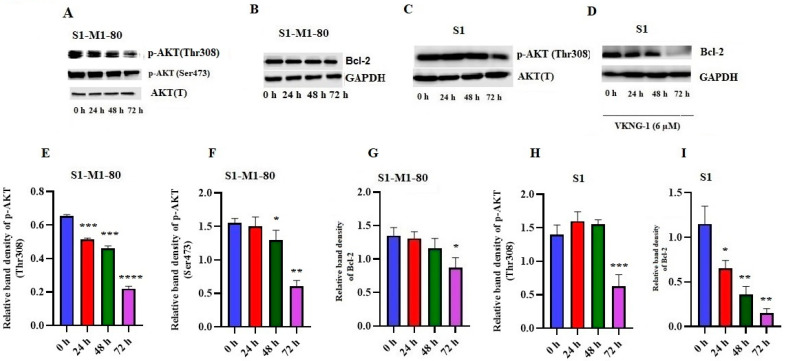
Effect of VKNG-1 on the expression of p-AKT and BCl-2 in S1 and S1-M1-80 cells: The relative intensity of p-AKT (Thr308 and Ser473) (**A**) and Bcl-2 (**B**) in S1-M1-80 cells and p-AKT (Thr308) (**C**) and Bcl-2 (**D**) in S1 cells after treatment with 6 μM of VKNG-1 for 24, 48 and 72 h. Relative quantification of the effect of VKNG-1 on p-AKT (Thr308) (**E**), Ser473 (**F**) and Bcl-2 (**G**) in S1-M1-80 cells and Thr308 (**H**) and Bcl-2 (**I**) in S1 cell line. The expression level of the target protein, Bcl-2 was normalized to GAPDH and the levels of p-AKT (Thr308 and Ser473) were normalized to AKT(T). Equal amounts of total cell lysates were employed for each sample and Western blot analysis was conducted. * *p* < 0.05, ** *p* ≤ 0.01, *** *p* ≤ 0.001 and **** *p* ≤ 0.0001 compared to the control group.

Previously, a study showed that the activation of the PI3K/AKT pathway increases the ABCG2 protein expression, thereby increasing the likelihood of MDR in certain cancer cells [34]. Therefore, we established the effect of VKNG-1 on the inhibition of the phosphorylated proteins, p-AKT and Bcl-2 in S1-M1-80 cancer cells using Western blotting. The incubation of S1-M1-80 cells with 6 µM of VKNG-1 for 72 h significantly reduced p-AKT activation (Ser473 and Thr308) (Figure 6A,E,F) and the downstream protein, Bcl-2 (Figure 6B,G)

To establish whether VKNG-1 effect on ABCG2 protein is by a direct inhibition on p-AKT protein activation which in turn can regulate the expression of ABCG2, a Western blotting was performed on parental S1 cells. VKNG-1 at 6 µM significantly decreased the activation of p-AKT(Thr308) (Figure 6C,H) and Bcl-2 proteins (Figure 6D,I) when these cells were treated for 24, 48 and 72 h.

### 2.7. Effect of VKNG-1 on Ras, NF-kB p65, PI3K p110α and 110β Expression

**Figure 7:**
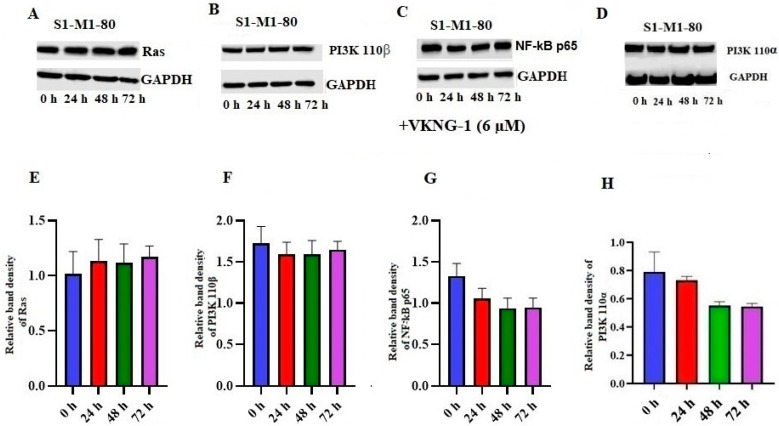
Effect of VKNG-1 on the expression of Ras, PI3K 110α,β and NF-kB p65: The relative intensity of Ras (**A**), PI3K 110β (**B**), NF-kB p65 (**C**) and PI3K 110α (**D**) was tested after the cells were treated with 6 μM of VKNG-1 for 24, 48 and 72 h. Relative quantification of the effect of VKNG-1 on Ras (**E**), PI3k 110β (**F**) and NF-kB p65 (**G**) and PI3K 110α (**H**) in S1-M1-80 cells. The expression levels of the target proteins, Ras, PI3K 110α and β and NF-kB p65 were normalized to GAPDH. Equal amounts of total cell lysates were employed for each sample and Western blot analysis was conducted.

To find out the effect of VKNG-1 on other target proteins, we established the effect of VKNG-1 on the expression of Ras, NF-kB p65 and PI3K p110α and β activation/inhibition in S1-M1-80 cells using Western blotting. The incubation of S1-M1-80 cells with 6 µM of VKNG-1 for 72 h could not significantly change Ras (Figure 7A,E), PI3K p110α and β (Figure 7B,D,F,H) and NF-kB p65 (Figure 7C,G) expression, compared to the vehicle.

### 2.8. VKNG-1 Reverses ABCG2-Mediated MDR in a Nude Mouse Xenograft Model

**Figure 8:**
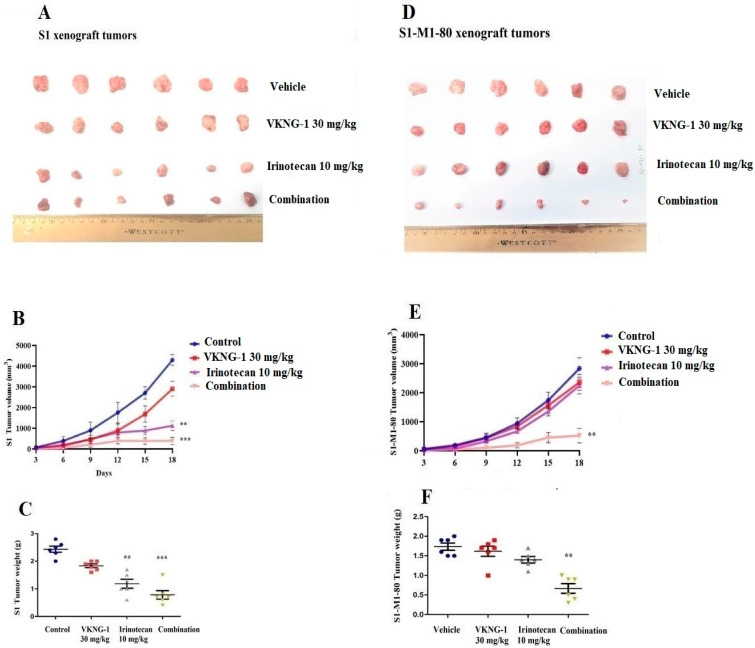
VKNG-1 inhibits ABCG2 activity in vivo. Effect of VKNG-1 on parental S1 and drug resistant S1-M1-80 cells. NCR nude mice were injected with S1 parental (**A**–**C**) and S1-M1-80 drug resistant (**D**–**F**) cells subcutaneously. During the treatment period, irinotecan significantly inhibited the S1 tumor xenograft growth compared to vehicle and VKNG-1 treated groups and combination of VKNG-1 and irinotecan significantly suppressed the growth of S1-M1-80 tumor xenografts compared to control and irinotecan alone group. Values indicate mean ± SD of six mice per group. Similar results were seen in two independent experiments and one result was used as a representative. ** *p* ≤ 0.01 and *** *p* < 0.001 compared to the control group.

**Figure 9:**
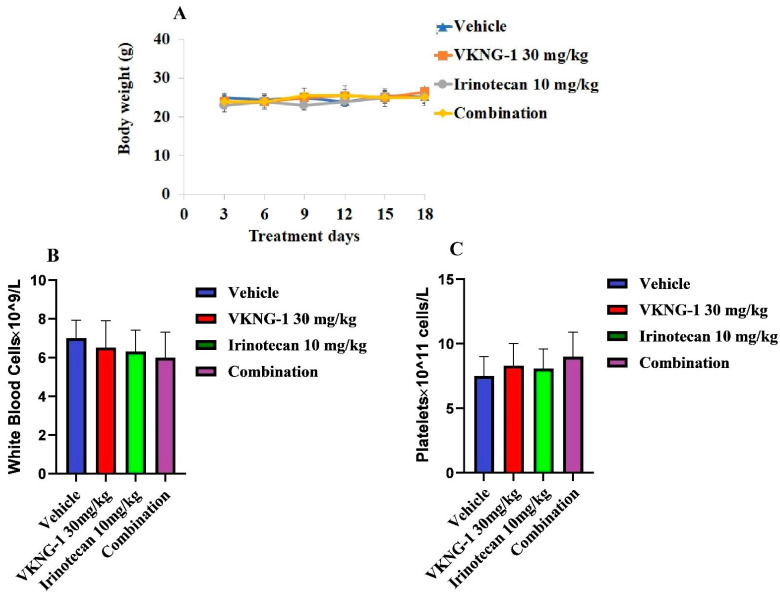
Changes in mean body weight before and after treatment for xenograft model are shown (**A**). The differences in mean white blood cells in nude mice (*n* = 6) at the end of the 18-day treatment period (**B**) The changes in mean platelets in nude mice (*n* = 6) at the end of the 18-day treatment period (**C**).

Parental cells, S1, and drug-resistant cells, S1-M1-80, were implanted subcutaneously and over a period of 7 to 10 days, the mice developed visible tumors and subsequently, the treatment regimen was started. The mice implanted with the S1 parental xenografts, which lack the ABCG2 transporter, had a significant reduction in weight and volume of the tumor after treatment with 10 mg/kg i.p. of irinotecan compared to the control group (Figure 8B,C). However, there was no notable change in the size of tumors in the group receiving oral VKNG-1 (30 mg/kg) (Figure 8A). The administration of 10 mg/kg i.p. of irinotecan did not significantly decrease the size or weight of the drug-resistant xenograft tumors (Figure 8D). In contrast, there was a remarkable decrease in tumor growth and weight in mice given 10 mg/kg i.p. of irinotecan and 30 mg/kg oral VKNG-1 compared to the control group (Figure 8E,F), which showed that VKNG-1 could enhance the anticancer effect of irinotecan, a ABCG2 transporter substrate. Importantly, the doses that we administered suggested that the combination treatment did not cause noticeable toxicity as there was no mortality or a significant loss in body weight (Figure 9A) and no significant change in blood cell count (Figure 9B,C).

## 3. Discussion

The occurrence of drug resistance is a serious issue in the treatment of cancer [4]. The advantage of ABC inhibitors is that they can restore anticancer drug efficacy in resistant tumors, although certain ABC inhibitors can produce significant adverse effects and toxicity [35]. VKNG-1 has been identified as a novel ABCG2 inhibitor that has a favorable preclinical toxicity profile that can restore the in vitro and in vivo efficacy of chemotherapeutic drugs that are ABCG2 substrates in drug-resistant colon cancers (see Table 1 and Table 2). Although HEK293/ABCG2 transfectant cells express relative low level of ABCG2, and it seems that ABCG2 upregulation is the single factor for drug resistance, it is surprised that reversal effects of VKNG-1 on HEK/ABCG2 transfectant cells is not as good as that on the S-1-M1-80, a drug selected resistant cell line. An efficient inhibitor is a compound that achieves full (90–100%) reversal of drug efflux at a concentration that does not exhibit significant off-target toxicity in vitro. In a previous study, human leukemia K562 cells expressing various levels of ABCB1 were used and cells expressing higher ABCB1 levels required higher concentrations of imatinib and nilotinib to achieve full reversal of drug efflux [36]. It has been showed that resistance to anticancer drugs is proportional to the expression level of the ABC transporters. For example, the resistance to imatinib in the human leukemia cell line (K562) was proportional to the expression level of ABCG2. However, K562/ABCG2-Z cell line with the lowest expression of ABCG2 significantly decreased the intracellular level of nilotinib, thereby mediating significant resistances to this drug [37]. It is not easy to establish correlation between the ABC transporter gene expression and the transporter activity. mRNA level of ABCG2 gene provides neither relevant information about its protein level nor about its function. For example, K562 cells express a very high level of the ABCB1 mRNA when compared and based on these data one could expect a significant ABCB1-mediated resistance in K562 cells. However, K562 cells do not express a detectable function of the ABCB1 transporter and level of ABCB1 gene at protein level is below detection limits [38]. It has been studied that gene activation might be a more common means of developing resistance than gene amplification. It seems likely that more than one mechanism contributes to the process of MDR. The ability to identify a group of tumors that display multidrug resistance by measuring the levels of MDR mRNA and its activity could have many important consequences. The information may be useful in designing or altering chemotherapeutic protocols in these patients [39,40].

Our results established that the reversal efficacy of VKNG-1 was selective for the ABCG2 transporter, as it did not have a reversal effect on ABCB1, ABCC1 and ABCC10 transporters (Table 3, Table 4 and Table 5). Moreover, VKNG-1 has a better toxicity profile as it is non-toxic on CCD-18Co cells even at high doses. Furthermore, at 10 µM, VKNG-1 significantly enhanced the accumulation of (^3^H)-mitoxantrone in the drug-resistant S1-M1-80 cancer cells, whereas accumulation of (^3^H)-mitoxantrone in the S1 cancer cell line, was not significantly altered by VKNG-1. This increase in mitoxantrone accumulation could result from either VKNG-1 blocking the pumping out action of the ABCG2 transporter and/or increasing the cellular uptake of mitoxantrone. Our results indicated that VKNG-1 caused a remarkable, time-dependent rise in the levels of (^3^H)-mitoxantrone. Given that this increase could have resulted from an inhibition of the ABCG2 transporter and/or downregulation of ABCG2 protein, Western blotting and PCR experiments were performed to determine the protein and mRNA expression, respectively, in the drug-resistant cell line. The data demonstrated that the treatment of drug-resistant S1-M1-80 cancer cells and ABCG2 transfected HEK293/R2 cells with 6 µM of VKNG-1 for 72 h notably diminish the ABCG2 protein expression in comparison with the vehicle. The reversal efficacy of VKNG-1 in S1-M1-80 cancer cells is because of its blockade of the ABCG2 transporter efflux function and downregulation of the ABCG2 protein. There is a possibility that VKNG-1 could affect the trafficking of the ABCG2 protein from the endoplasmic reticulum to the cell membrane, thus decreasing the number of ABCG2 transporter available for drug efflux. Consequently, we performed in vitro immunofluorescent experiment to evaluate the cellular localization of the ABCG2 transporter protein. The results indicated that 6 µM of VKNG-1 did not cause a change in the location of the ABCG2 protein in the S1-M1-80 cells compared to incubation with the vehicle. Previously, it has been shown that catalytic subunits of phosphatidylinositol 3-kinase (PI3K) are involved in ABC transporter-mediated MDR and the subunits, P110α or P110β [41], may represent specific targets for reversing MDR cancer. The inhibition of these subunits of PI3K with the PI3K inhibitor, BAY-1082439, downregulates the ABCG2 transporter expression and reverses resistance to mitoxantrone in non-small cell lung cancer (NSCLC), H460/MX20 MDR cells [34]. In addition, another study showed that the inhibition of the protein, p-AKT, significantly decreased ABCG2-mediated MDR [42]. Since VKNG-1 significantly downregulated the ABCG2 expression at the mRNA and protein levels, we conducted studies to determine the possible mechanism by which VKNG-1 decreases MDR. VKNG-1, at 6 µM, significantly inhibited p-AKT and Bcl-2 protein expression in the S1-M1-80 cancer cells, suggesting that VKNG-1, in part, reverses ABCG2-mediated drug resistance by decreasing the activation of p-AKT and Bcl-2 without changing Ras, AKT(T), NF-kB p65 expression and P110α, 110β activation. Abnormal activation of the PI3K/AKT pathway counteracts the chemotherapeutic-induced apoptosis via the enhancing of anti-apoptotic genes, such as Bcl-2, and reducing of pro-apoptotic genes to modulate MDR. Resistant cells express higher levels of Bcl-2 and overexpression of Bcl-2 typically results in cancer cell resistance which is associated with abnormal changes in PI3K/AKT pathways. In summary, activation of the PI3K/AKT pathway promotes MDR via the regulation of a variety of cellular apoptotic processes [43]. The effect of VKNG-1 on the expression of ABCG2 protein is a result of direct inhibition of p-AKT can be further proved by a Western blotting experiment conducted on parental S1 cells which shows a significant downregulation of p-AKT and Bcl-2 protein. Some previous studies suggest that AKT signaling pathway is involved in retaining the ABCG2 protein in the plasma membrane, while an inhibition of this pathway may cause the appearance of the transporter in intracellular vesicles with enhanced recycling [44]. Since AKT phosphorylation is inhibited by VKNG-1, the inhibition of this pathway may be involved in ABCG2 internalization and degradation. To find out association between p-AKT and ABCG2 down-regulation, a Western blotting was performed on S1-M1-80 cells treated with p-AKT inhibitor, gilteritinib at a concentration of 3 µM for 24, 48 and 72 h and a also a combination of gilteritinib and VKNG-1 for 72 h. Gilteritinib, a small molecule, is an inhibitor of FMS-like tyrosine kinase 3 (FLT3) and is approved by FDA for treating acute myeloid leukemia (AML). It has demonstrated that gilteritinib induces cell growth inhibition and caspase-dependent apoptosis in CRC cells. It also results in the inhibition of AKT activation and combination of gilteritinib with other anticancer drugs, such as 5-FU, lead to robust induction of apoptosis in CRC cells [45]. The results showed that treatment with both gilteritinib alone (72 h) and a combination of both drugs remarkably downregulated ABCG2 mRNA and protein levels which suggest that ABCG2 inhibition can be related to the inhibition of phosphorylated AKT. It is shown that AKT signaling is the most activated signaling pathway in 57% of all colorectal cancers and activation of AKT is an early event in colon carcinogenesis. AKT phosphorylation at Thr308 and Ser473 were detected in colon carcinomas and AKT activation may be important in the early inhibition of apoptosis during colon carcinogenesis [46]. In addition, previous studies suggested that PI3K/AKT promotes ABCG2 transcription mainly through Kelch-like ECH-associated protein 1 (KEAP1) and nuclear factor erythroid-derived (Nrf2) pathway which are downstream signals to AKT pathway that might also cause the ABCG2-mediated chemoresistance [47].

Given our in vitro findings, molecular docking was performed to assess the interaction of VKNG-1 with the ABCG2 transporter using a human ABCG2 model. The docking score we obtained for VKNG-1 was −12.929 kcal/mol, which is much lower than that of other known ABCG2 inhibitors like bafetinib (−9.66 kcal/mol) [48] and voruciclib (−10,304 kcal/mol). The docking score and the magnitude of the interaction between the inhibitor and ABCG2 are inversely related [49] and it indicates that VKNG-1 interacts with the ABCG2 transporter to a great extent compared to other ABCG2 inhibitors such as bafetinib, VS-4718, voruciclib and cabozantinib.

Finally, based on our in vitro results, in vivo study was conducted to determine if the systemic administration of VKNG-1 would increase the efficacy of the anticancer drug, irinotecan, in mice implanted with cancer cells overexpressing the ABCG2 transporter. There were no notable differences in tumor size, weight and volume between the control, irinotecan, and VKNG-1 alone treated groups in mice xenografted with S1-M1-80 cancer cells. However, VKNG-1 treatment (combination with irinotecan) restored the anticancer efficacy of irinotecan compared to the irinotecan alone group. Moreover, the oral administration of 30 mg/kg of VKNG-1 with irinotecan (10 mg/kg i.p.) remarkably attenuated the tumor growth in mice implanted with ABCG2 overexpressing S1-M1-80 mouse xenografts. Meanwhile, there was no notable change in body weight, WBC, and platelets count, suggesting that VKNG-1 can be tolerated at this dose and may be a promising candidate for treating tumors that overexpress the ABCG2. Our in vivo results suggest that VKNG-1 significantly increases the anticancer efficacy of irinotecan in mice with S1-M1-80 cancer cell xenografts at a dose that does not produce significant toxic effects.

## 4. Materials and Methods

### 4.1. Chemicals

VKNG-1 (N-(2-(2-(3-methoxyphenyl)-2H-tetrazol-5-yl) phenyl)-4-aminobenzamide) was synthesized as previously described [25]. Mitoxantrone and doxorubicin were received from Medkoo Biosciences (Morrisville, NC, USA). 7-Ethyl-10-hydroxycamptothecin (SN-38), cisplatin, verapamil, vincristine, paclitaxel, irinotecan, MTT (3-(4,5-dimethylthiazol-yl)-2,5-diphenyltetrazolium bromide), and dimethylsulfoxide were bought from Sigma Chemical (St. Louis, MO, USA). Fumitremorgin C (FTC) was procured from the laboratory of Thomas McCloud, NIH (Bethesda, MD, USA). Cepharanthine was obtained from Dr. Akiyama (Kagoshima University, Korimoto 1-21-24, Kagoshima 890-8580, Japan) and Gilteritinib was received from Chemietek (Indianapolis, IN, USA). MK-571 was obtained from EMD Millipore (Philadelphia, PA, USA). Dulbecco’s modified Eagle’s medium, 0.25% trypsin, antibiotic, penicillin/streptomycin (P/S) and FBS were obtained from Hyclone (Waltham, MA, USA). Tritium labeled mitoxantrone was acquired from Moravek Biochemicals, Inc. (Brea, CA, USA). The monoclonal antibodies D16H11 (against GAPDH), Ser473 D9E (against p-AKT), C73F8 (against PI3k p110α), D25E6 (against p-AKT Thr308), 67648T (against Ras), C33D4 (against PI3K p 110β) and D14E12 (against NF-kB p65) were obtained from Cell Signaling (Danvers, MA, USA). BXP-21 (against ABCG2) and Alexa flour conjugated secondary antibody were obtained from Santacruz Biotechnology, TX, USA. The ABCG2 and 18S TaqMan gene expression kits and superscript IV reverse transcription kit were obtained from Fisher Scientific (Waltham, MA, USA).

### 4.2. Cell Lines

CCD-18Co (human colon fibroblast cell line), S1 (human colon cancer parental cell line) and the mitoxantrone selected ABCG2-overexpressing drug-resistant subline, S1-M1-80, were used for the ABCG2 reversal experiments. SW620, the colon cancer cell line (human) and SW620/AD300, the drug-resistant cell line which highly expresses ABCB1 transporter were employed for the ABCB1 reversal experiments. They were cultured and maintained in DMEM medium supplemented with 10% FBS and 1% P/S and maintained in 5% CO_2_ at 37 °C. Drug-resistant cells, S1-M1-80 was grown in the DMEM medium which has the anticancer drug, mitoxantrone, gradually increasing its concentration up to 80 µg/mL, inducing the overexpression of the ABCG2 transporter [50]. The drug-resistant, SW620/AD300 cells were grown with a concentration of 300 ng/mL of doxorubicin [51]. For the ABCG2 reversal experiments, we used the transfected cell lines, HEK293/pcDNA3.1 (human embryonic kidney cell line transfected with empty vector), HEK293 cells transfected with ABCG2 transporter which are HEK293/R482 (wild-type) and two variants, HEK293/R482G and HEK293/R482T [52]. HEK293 cells were grown after selecting them with G418, at a concentration of 2000 µg/mL, after transfecting the parental HEK293 cells with an empty pcDNA3.1 vector (HEK293/pcDNA3.1) or pcDNA3.1 vector containing the ABCG2 DNA coding for either arginine (R) (HEK293/R482), glycine (G) (HEK293/R482G) or threonine (T) (HEK293/R482T) at position 482. The parental cell line, HEK293/pcDNA3.1 was transfected with the ABCC1 and ABCC10 transporters DNA to generate HEK293/ABCC1 and HEK293/ABCC10 cells, respectively [53,54], to find out the reversal activity of VKNG-1 on ABCC1 and ABCC10. SW620 and SW620/AD300 cells, S1 and ABCG2-overexpressing S1-M1-80 cells were kindly provided by Dr. Susan Bates (Columbia University, NY, USA) and HEK/pcDNA3.1, HEK293/R482, HEK293/R482G and HEK293R482T were obtained from Robert Robey (NCI, NIH, Bethesda, MD, USA). HEK293/ABCB1 was kindly provided by Dr. Suresh V. Ambudkar (NCI, NIH, Bethesda, MD, USA). The fibroblast cell line, CCD-18Co was purchased from ATCC (Manassas, VA, USA).

### 4.3. Experimental Animals

Athymic (nu/nu) nude mice (male, 20–26 g, around 6 weeks of age) were bought from the Taconic Farms (Albany, NY, USA). The mice were kept under alternate light/dark cycles, provided with food and water, and kept in separate cages. They were housed at the St. John’s University Animal Care Center and regularly watched for the growth of tumors using vernier calipers. The animal protocol was already accepted by the Institutional Animal Care and Use Committee (IACUC). This study was conducted carried out in compliance with the Animal Welfare Act and the National Institutes of Health guide for the care and use of laboratory animals.

### 4.4. Determination of Cytotoxicity by MTT Assay

It was conducted in a 96 well plate using CCD-18Co, parental and drug-resistant cell lines that were seeded at a density of 4 × 10^3^ cells/well. Following 24 h incubation, the cells were treated with VKNG-1 (ranging from a concentration of 0–100 µM). After 68 h, 20 µL of 4 mg/mL MTT, was added to each well and the plates were further incubated 37 °C for 4 h. Subsequently, the MTT was removed from all wells and 100 µL of DMSO was added to dissolve the formazan crystals formed by the reduction of MTT in the mitochondria of the viable cells. The absorbance was measured at 570 nm using a spectrophotometer (Thermo Fisher Scientific, Waltham, MA, USA) by a 3-(4,5-dimethylthiazol-2-yl)-2,5-diphenyl-tetrazolium bromide (MTT) calorimetric assay as previously described [55].

The least toxic concentration of VKNG-1 for the reversal study was determined. Based on the cytotoxicity data, the safe concentrations at which survival of around 85% of parental and resistant cells (1 and 6 µM) were selected for the reversal experiments.

### 4.5. Screening of VKNG-1 for ABC Transporters Reversal Activity

Once the cells were attached, the chemotherapeutic drugs were added at different concentrations (20 µL/well) into the designated wells after incubating for 2 h with VKNG-1 (1 and 6 µM), which was the same concentration of the other positive controls: FTC (6 µM) for ABCG2, verapamil (5 µM) for ABCB1, MK571 (25 µM), which is an ABCC1 inhibitor and cepharanthine (5 µM), an ABCC10 inhibitor. After 68 h, the MTT assay was conducted, and absorbance was measured [55].

### 4.6. (^3^H)-Mitoxantrone Accumulation and Efflux Assay

The intracellular accumulation of radioactive labeled (^3^H)-mitoxantrone and the efflux activity of the ABCG2 transporter (at 0, 30, 60 and 120 min), in the parental (S1) and resistant (S1-M1-80) cells, was calculated with or without the inhibitor [56]. The cells were cultured in DMEM in the presence or absence of reversal compound (VKNG-1 at 1 and 10 µM and FTC at 10 µM) at 37 °C for 2 h. Subsequently, the cells were treated with 0.01 µM (^3^H)-mitoxantrone in the presence or absence of the inhibitor at 37 °C for another 2 h. The radioactivity was quantified with a scintillation counter from Packard Instrument Company, Inc. (Downers Grove, IL, USA).

### 4.7. Western Blot Analysis

The ABCG2, p-AKT, Ras, Bcl-2, NF-kB p65, AKT(T) and phosphoinositide-3-kinase catalytic subunits (PI3K p110α and β) protein levels were measured using Western blotting in the presence of VKNG-1 (6 µM) and gilteritinib (3 µM) for 24, 48 and 72 h, using a previously described method [48] and quantified using ImageJ. The relative expression of all target genes is normalized to the house keeping gene, GAPDH.

### 4.8. mRNA Expression

S1 and S1-M1-80 cancer cell lines were treated with 6 µM of VKNG-1 for 24, 48, and 72 h and the total amount of RNA was extracted by the RNA extraction trizol reagent as previously described [57]. Ultrapure, intact RNA is essential for high quality cDNA synthesis and important for accurate mRNA quantification. RNA should be devoid of any RNAase contamination, and aseptic conditions should be maintained. RNA was quantified using Nanodrop and RNA samples with a A260/280 ratio in the range of 1.8 to 2.0 were subjected to reverse transcription and the cDNAs formed (by superscript IV reverse transcription kit) (1 µg) were used for quantitative PCR. The PCR data were quantitated using the ∆∆Ct method and presented as relative-fold of mRNA expression. The threshold cycle (Ct) is the cycle number at which the fluorescent signal of the reaction crosses the threshold. The Ct value is inversely related to the starting amount of target. ∆∆Ct is the difference in Ct value of the gene-of-interest (ABCG2) and Ct value of the house keeping gene (18S).

### 4.9. Immunofluorescence

For the immunofluorescence experiments, the parental and drug-resistant cells were cultured with or without the inhibitor drug, VKNG-1 (6 µM) for 24, 48, and 72 h. The immunofluorescence was determined as stated before [58] and the green color represents ABCG2 expression on the cell membrane and red dye, propidium iodide was used to stain the nucleus.

### 4.10. Docking Analysis

Molecular modeling was performed using the software, Maestro 11.5 (Schrodinger, New York) as previously reported [59]. Ligand preparation and the human ABCG2 (PDB ID: 6FFC) [60] protein preparation was carried out, followed by forming a grid in the binding pocket of ABCG2. Glide XP docking and induced-fit docking were conducted using the protocol.

### 4.11. MDR Mouse Xenograft Models

The drug-resistant S1-M1-80 mouse xenograft models were established as reported earlier [61]. Parental, S1 (5 × 10^6^) and S1-M1-80 (1 × 10^7^) cells were implanted subcutaneously into immunocompromised mice under the left and right armpit regions, respectively under 3% isoflurane anesthesia. The different cell numbers of S1 and S1-M1-80 were chosen based on a pilot study that was conducted by our laboratory at St. John’s University. The parental S1 cells grow faster than drug-resistant S1-M1-80 cells which account for implanting different cell numbers. When the diameter of the tumors was around 0.4 cm, the mice were grouped into four different treatment groups consisting of six mice per group as follows: (a) polyethylene glycol 300 as the vehicle which was given orally (q3d × 6), (b) irinotecan (10 mg/kg, q3d × 6) was given intraperitoneally (i.p.), dissolved in normal saline [62] (c) VKNG-1 (30 mg/kg) was diluted in PEG 300 and given orally (q3d × 6) (based on the pilot study) and (d) the combination group was given 30 mg/kg of VKNG-1 (q3d × 6) orally, 1 h before irinotecan (10 mg/kg, i.p., q3d, six times). The treatment was given for 18 days, and the body weights were noted every other day to calculate the drug dosage. Tumor volumes (calculated using the length (A) and width (B) of the tumor) were calculated every third day using vernier calipers and calculated using the following previously published formula, (V = π/6 (A + B/2)^3^) [63]. The blood was taken via submandibular puncture on the last treatment day and white blood cells (WBC) and platelet counts were determined in all four groups.

After finishing the treatment regimen, the mice were euthanized, and S1 and S1-M1-80 tumors were removed, and tumor weights were noted.

### 4.12. Statistical Analysis

All experiments were performed a minimum of three times and the differences were calculated using GraphPad Prism (version 8) and analyzed using either a one-way or two-way ANOVA, followed by Dunnett’s post hoc analysis. The a priori statistical significance value was *p* < 0.05.

## 5. Conclusions

The results of our study show that the novel compound, VKNG-1, significantly reverses MDR in S1-M1-80 cancer cells by blocking the efflux activity of the ABCG2 transporter and downregulating ABCG2 and p-AKT protein. VKNG-1, at a dose of 30 mg/kg p.o., did not produce any noticeable toxic effects in the in vivo tumor xenografted mice. The combination of 30 mg/kg oral VKNG-1 with a dose of 10 mg/kg i.p. of irinotecan significantly reduced tumor size, growth rate, and tumor volume in mice with ABCG2-overexpressing tumor xenografts, compared to either drug alone in ABCG2-overexpressing colon cancers.

## Figures and Tables

**Table 1:** The effect of VKNG-1 on the reversal of ABCG2-mediated MDR.

Cell Lines	S1		S1-M1-80	
	IC_50_ ± SD (µM)	FR	IC_50_ ± SD (µM)	FR
Mitoxantrone	0.147 ± 0.013	(1.0)	23.321 ± 2.602	(151.7)
+VKNG-1 (1 µM)	0.117 ± 0.018	(0.8)	0.771 ± 0.149	(4.7)
+VKNG-1 (6 µM)	0.115 ± 0.019	(0.8)	0.268 ± 0.055	(1.6)
+FTC (6 µM)	0.129 ± 0.013	(0.9)	0.175 ± 0.033	(1.2)
SN-38	0.113 ± 0.071	(1.0)	25.867 ± 2.884	(228.2)
+VKNG-1 (1 µM)	0.153 ± 0.053	(1.3)	0.889 ± 0.127	(5.6)
+VKNG-1 (6 µM)	0.117 ± 0.024	(1.0)	0.245 ± 0.092	(2.2)
+FTC (6 µM)	0.121 ± 0.020	(1.0)	0.197 ± 0.025	(1.7)
Doxorubicin	0.139 ± 0.022	(1.0)	9.579 ± 1.534	(73.1)
+VKNG-1 (1 µM)	0.083 ± 0.011	(0.6)	0.581 ± 0.097	(4.5)
+VKNG-1 (6 µM)	0.165 ± 0.015	(1.2)	0.063 ± 0.013	(0.5)
+FTC (6 µM)	0.125 ± 0.019	(0.9)	0.193 ± 0.032	(1.5)
Cisplatin	154.959 ± 22.426	(1.0)	194.845 ± 24.119	(1.3)
+VKNG-1 (1 µM)	150.539 ± 24.745	(0.9)	180.931 ± 32.527	(1.2)
+VKNG-1 (6 µM)	159.259 ± 18.131	(1.1)	173.985 ± 30.406	(1.1)
+FTC (6 µM)	153.51 ± 34.951	(1.2)	195.785 ± 23.335	(1.3)

µM-Micromole; Values in tables indicate at least three independent experiments performed in triplicates. IC_50_: concentration of the drug that is required for inhibition of cell survival by 50% (mean ± SD). FR: Fold of Resistance was calculated by dividing the IC_50_ value of an anticancer drug in drug-resistant S1-M1-80 cells in the presence or absence of an inhibitor (VKNG-1 or FTC) by the IC_50_ value of parental S1 cells without an inhibitor (VKNG-1 or FTC).

**Table 2:** The reversal effect of VKNG-1 on the cytotoxicity of anticancer drugs in ABCG2 transfected cell lines.

Cell Lines	HEK293/pcDNA3.1		HEK293/R2		HEK293/G2		HEK293/T7	
	IC_50_ ± SD (µM)	FR	IC_50_ ± SD (µM)	FR	IC_50_ ± SD (µM)	FR	IC_50_ ± SD (µM)	FR
Mitoxantrone	0.0180 ± 0.0001	(1.0)	0.7449 ± 0.0214	(41.3)	0.7111 ± 0.0536	(39.5)	0.6065 ± 0.0394	(33.6)
+VKNG-1 (1 µM)	0.0187 ± 0.0006	(1.0)	0.6827 ± 0.0572	(37.9)	0.3974 ± 0.0051	(22.1)	0.5542 ± 0.0306	(30.7)
+VKNG-1 (6 µM)	0.0182 ± 0.0008	(1.0)	0.1008 ± 0.0347	(5.5)	0.0600 ± 0.0172	(3.3)	0.1372 ± 0.0265	(7.6)
+FTC (6 µM)	0.0167 ± 0.0005	(0.9)	0.1428 ± 0.0377	(7.9)	0.0772 ± 0.0189	(4.3)	0.1482 ± 0.0206	(8.3)
SN-38	0.0129 ± 0.0007	(1.0)	0.7319 ± 0.0023	(56.7)	0.4932 ± 0.0012	(38.2)	0.4430 ± 0.0010	(34.3)
+VKNG-1 (1 µM)	0.0157 ± 0.0028	(1.2)	0.3090 ± 0.0377	(23.9)	0.1033 ± 0.0082	(8.0)	0.1314 ± 0.0169	(10.1)
+VKNG-1 (6 µM)	0.0113 ± 0.0007	(0.8)	0.0309 ± 0.0065	(2.3)	0.0534 ± 0.0047	(4.1)	0.0218 ± 0.0015	(1.7)
+FTC (6 µM)	0.0129 ± 0.0012	(1.0)	0.0355 ± 0.0050	(2.8)	0.0577 ± 0.0007	(4.5)	0.0233 ± 0.0030	(1.8)
Doxorubicin	0.0152 ± 0.0001	(1.0)	0.6225 ± 0.0332	(40.9)	0.5994 ± 0.0054	(39.3)	0.6858 ± 0.0367	(44.9)
+VKNG-1 (1 µM)	0.0154 ± 0.0001	(1.0)	0.2395 ± 0.0257	(15.7)	0.2345 ± 0.0213	(15.3)	0.2222 ± 0.0407	(14.5)
+VKNG-1 (6 µM)	0.0157 ± 0.0001	(1.0)	0.0662 ± 0.0004	(4.3)	0.0597 ± 0.0073	(3.9)	0.0752 ± 0.0052	(4.9)
+FTC (6 µM)	0.0180 ± 0.0001	(1.2)	0.0771 ± 0.0105	(5.1)	0.0694 ± 0.0024	(4.6)	0.0817 ± 0.0097	(5.3)
Cisplatin	1.8455 ± 0.2609	(1.0)	1.8567 ± 0.0598	(1.0)	1.7348 ± 0.0379	(0.9)	1.9224 ± 0.0543	(1.0)
+VKNG-1 (1 µM)	1.7990 ± 0.2319	(0.9)	1.8607 ± 0.0464	(1.0)	1.9977 ± 0.0471	(1.1)	1.9400 ± 0.1171	(1.0)
+VKNG-1 (6 µM)	1.8835 ± 0.0955	(1.0)	1.9156 ± 0.0896	(1.0)	1.7695 ± 0.0213	(0.9)	1.9864 ± 0.1278	(1.1)
+FTC (6 µM)	1.9950 ± 0.1245	(1.0)	2.0023 ± 0.1251	(1.1)	1.7413 ± 0.0731	(0.9)	1.8896 ± 0.0611	(1.0)

µM-Micromole. Values in Table 2 indicate least three independent experiments performed in triplicates. IC_50_: concentration of the drug that is required for inhibition of cell survival by 50% (mean ± SD). FR: Fold of Resistance was calculated by dividing the IC_50_ value of an anticancer drug in an ABCG2 plasmid transfected cell subline in the presence or absence of an inhibitor by the IC_50_ value of the empty vector transfected cell line without an inhibitor.

**Table 3:** The reversal effect of VKNG-1 on the cytotoxicity of anticancer drugs in ABCB1- overexpressing cells.

Cell Lines	SW620		SW620/AD300	
	IC_50_ ± SD (µM)	FR	IC_50_ ± SD (µM)	FR
Doxorubicin	0.083 ± 0.012	(1.0)	9.371 ± 1.45	(117.1)
+VKNG-1 (1 µM)	0.101 ± 0.014	(1.2)	9.321 ± 1.67	(112.3)
+VKNG-1 (5 µM)	0.098 ± 0.011	(1.1)	8.759 ± 1.89	(105.5)
+Verapamil (5 µM)	0.094 ± 0.013	(1.1)	1.056 ± 0.153	(13.2)

µM-Micromole. Values in Table 3 indicate at least three independent experiments performed in triplicates. IC_50_: concentration of the drug that is required for inhibition of cell survival by 50% (mean ± SD). FR: Fold of Resistance was calculated by dividing the IC_50_ value of an anticancer drug in drug-resistant cell line SW620/AD300 in the presence or absence of an inhibitor by the IC_50_ value of parental SW620 cells without an inhibitor.

**Table 4:** The reversal effect of VKNG-1 on the cytotoxicity of anticancer drugs in ABCC1-transfected cell line.

Cell Lines	HEK293/pcDNA3.1		HEK293/ABCC1	
	IC_50_ ± SD (µM)	FR	IC_50_ ± SD (µM)	FR
Vincristine	0.037 ± 0.007	(1.0)	1.169 ± 0.225	(30.5)
+VKNG-1 (1 µM)	0.035 ± 0.005	(0.9)	0.959 ± 0.153	(24.3)
+VKNG-1 (5 µM)	0.049 ± 0.005	(1.3)	0.811 ± 0.147	(21.9)
MK-571 (25 µM)	0.025 ± 0.002	(0.7)	0.225 ± 0.047	(6.1)

µM-Micromole. Values in Table 4 indicate at least three independent experiments performed in triplicates. IC_50_: concentration of the drug that is required for inhibition of cell survival by 50% (mean ± SD). FR: Fold of Resistance was calculated by dividing the IC_50_ value of an anticancer drug in a gene transfected cell line in the presence or absence of an inhibitor by the IC_50_ value of empty vector transfected HEK239 cell line without an inhibitor.

**Table 5:** The reversal effect of VKNG-1 on the cytotoxicity of anticancer drugs in ABCC10-transfected cell line.

Cell Lines	HEK293/pcDNA3.1		HEK293/ABCC10	
	IC_50_ ±SD (µM)	FR	IC_50_ ±SD (µM)	FR
Paclitaxel	3.523 ± 0.539	(1.0)	53.356 ± 4.33	(15.1)
+VKNG-1 (1 µM)	3.213 ± 0.471	(0.9)	50.891 ± 6.67	(14.3)
+VKNG-1 (5 µM)	3.888 ± 0.613	(1.3)	45.323 ± 5.33	(12.9)
Cepharanthine (5 µM)	3.789 ± 0.469	(1.1)	3.588 ± 0.519	(1.1)

µM-Micromole. Values in Table 5 indicate at least three independent experiments performed in triplicates. IC_50_: concentration of the drug that is required for inhibition of cell survival by 50% (mean ± SD). FR: Fold of Resistance was calculated by dividing the IC_50_ value of an anticancer drug in a gene transfected cell line in the presence or absence of an inhibitor by the IC_50_ value of empty vector transfected HEK239 cell line without an inhibitor.

## Data Availability

The data presented in this study are available on request from the corresponding author.
